# Circular RNA circ_0020014 contributes to osteoarthritis progression via miR-613/ADAMTS5 axis

**DOI:** 10.17305/bjbms.2021.6668

**Published:** 2022-02-28

**Authors:** Zirui Yu, Fei Cong, Wentao Zhang, Tao Song, Shihui Zhang, Renqi Jiang

**Affiliations:** Department of Orthopaedics, Xi’an Hong-Hui Hospital Affiliated to Medical College of Xi’an Jiaotong University, Xi’an, Shaanxi, China

**Keywords:** Osteoarthritis, circ_0020014, miR-613, ADAMTS5

## Abstract

Circular RNAs have been shown to be significant regulators in osteoarthritis (OA), whereas the functional effect of circ_0020014 in OA remains unclear. Our goal was to try and understand the underlying regulatory mechanism of circ_0020014 in OA. The cartilage tissue was obtained from OA patients and trauma patients. Interleukin-1β (IL-1β)-treated chondrocytes (CHON-001) were used as the *in vitro* cellular model for OA. The expression levels of circ_0020014, microRNA-613 (miR-613), and a disintegrin and metalloproteinase with thrombospondin motifs 5 (ADAMTS5) were examined by real-time quantitative polymerase chain reaction. The protein level was detected using the Western blot assay. Cell viability and apoptosis were measured by 3-(4, 5-dimethylthiazol-2-yl)-2, 5-diphenyl-2H-tetrazol-3-ium bromide and flow cytometry assays, respectively. The secretion of inflammatory cytokine was determined by enzyme-linked immunosorbent assay. Circ_0020014 was upregulated in OA cartilage tissues and IL-1β-treated CHON-001 cells, compared with that in healthy cartilage tissues and untreated cells. IL-1β treatment induced cell injury by promoting inflammation and apoptosis, and inhibiting cell viability and extracellular matrix accumulation in chondrocytes. Circ_0020014 knockdown significantly protected CHON-001 cells from IL-1β-induced cell dysfunction. MiR-613 was targeted by circ_0020014 and negatively regulated ADAMTS5 expression. In addition, miR-613 downregulation or ADAMTS5 overexpression partly lessened the protective effect of circ_0020014 knockdown on IL-1β-treated CHON-001 cells. Collectively, circ_0020014 acted as a miR-613 sponge to regulate ADAMTS5 expression, thereby protecting chondrocytes from IL-1β-induced inflammatory damage, which might be a novel diagnostic marker for OA.

## INTRODUCTION

Osteoarthritis (OA) is a common age-associated joint disorder that is characterized by progressive articular cartilage destruction and sclerotic pathological changes in the subchondral bone [1]. Chondrocytes are the unique cells in articular cartilage tissues, which play prominent functions in maintaining the homeostasis of the extracellular matrix (ECM) [2]. In addition, chondrocytes dysfunction and ECM degeneration were recognized as two important steps in the initiation and progression of OA [3,4]. The regulatory mechanisms of OA pathogenesis are complex and multifactorial. An in-depth knowledge of the pathogenic mechanism of OA is necessary to seek novel and effective therapeutic strategy for OA therapy.

Circular RNAs (circRNAs) are a group of noncoding RNAs with a covalently circular structure and generated by back-splicing [5]. CircRNAs have been confirmed to engage in various pathological processes, including OA [6]. Several dysregulated circRNAs, including circ_0020014, were identified by microarray analysis and had been suggested to be involved in the regulation of OA development [7]. Circ_0020014 is derived from the dual specificity phosphatase 5 (*DUSP5*) gene and located at chromosome 10 (112, 266, 692-112, 271, and 302). Herein, we investigated the regulatory mechanism of circ_0020014 in OA progression.

MicroRNAs (miRNAs) are a group of regulatory RNAs (19-22 nucleotides in length) [8]. Numerous studies have suggested that the deregulation of miRNAs is involved in multiple biological processes [9]. miR-613 has been confirmed to function as a novel tumor facilitator miRNA in human tumors, including glioma [10], hepatocellular carcinoma [11], and triple-negative breast cancer [12]. In addition, miR-613 was thought to mitigate IL-1β-treated CHON-001 cell injury by regulating fibronectin 1 expression [13]. However, the regulatory mechanism and functional roles of miR-613 in OA still need further investigation.

A disintegrin and metalloproteinase with thrombospondin motifs 5 (ADAMTS5) is a member of ADAMTS family and a major aggrecan-degrading enzyme in cartilage [14,15]. ADAMTS5 was confirmed to take part in OA progression and was considered as an underlying biomarker for OA treatment [16]. Under pathological conditions, ADAMTS5 expression was significantly elevated in interleukin-1β (IL-1β)-induced chondrocytes, which triggered ECM degradation [17]. Hence, ADAMTS5 played a significant role in OA pathogenesis.

In this study, we measured the expression patterns of circ_0020014, miR-613, and ADAMTS5 in OA cartilage specimens and IL-1β-induced CHON-001 cells. We also investigated the correlation between miR-613 and circ_0020014 or ADAMTS5, as well as the regulatory mechanism underlying circ_0020014 in regulating OA progression, hoping to provide novel therapeutic strategies for OA.

## MATERIALS AND METHODS

### Patient specimens

Thirty OA cartilage tissues were obtained from OA patients (9 male and 21 female patients; aged 52-78), who underwent total knee arthroplasty at Xi’an Hong-Hui Hospital Affiliated to Medical College of Xi’an Jiaotong University. Healthy cartilage tissues were collected from age- and gender-matched trauma patients with amputation. Patients with confirmed OA were included in the study. Patients were excluded if they had any arthritis other than primary OA, including secondary OA, gouty arthritis, or rheumatoid arthritis; if they had any inflammatory arthropathy or other significant medical disease; or if they had previous knee surgery. All specimens were quickly frozen after operation and then stored at −80°C for subsequent study. The informed consent was acquired from recruited individuals before surgery. This study was approved by the Ethics Committee of Xi’an Hong-Hui Hospital Affiliated to Medical College of Xi’an Jiaotong University.

### Cell line

Human chondrocytes (CHON-001; American type culture collection, Manassas, VA, USA) were cultured in Dulbecco’s Modified Eagle’s Medium (DMEM, Thermo, Waltham, MA, USA) containing 10% (v/v) fetal bovine serum (GE Healthcare, Logan, UT, USA) at 37°C in a humidified incubator with 5% CO_2_. For IL-1β treatment, chondrocytes CHON-001 were hatched with increasing concentrations of IL-1β (Invitrogen, Carlsbad, CA, USA) for 24 hours or incubated with 10 ng/mL of IL-1β for different time.

### Real-time quantitative polymerase chain reaction (RT-qPCR)

RNA was isolated by TRIzol reagent (Invitrogen) and quantified with a NanoDrop (Thermo). Next, complementary DNA (cDNA) was synthesized using First Strand cDNA Synthesis Kit (Aidlab, Beijing, China) or miRNA 1^st^ strand cDNA synthesis kit (Agilent Technologies, Santa Clara, CA, USA). RT-qPCR assay was administered with cDNA, specific primers, SYBR Green Real-Time PCR Master Mixes (Thermo), and nuclease-free water under the Thermal Cycler CFX6 System (Bio-Rad, Hercules, CA, USA). Data were calculated using 2^-ΔΔCt^ method with GAPDH or U6 as internal control. The primer sequences are displayed in Table 1.

**TABLE 1:**
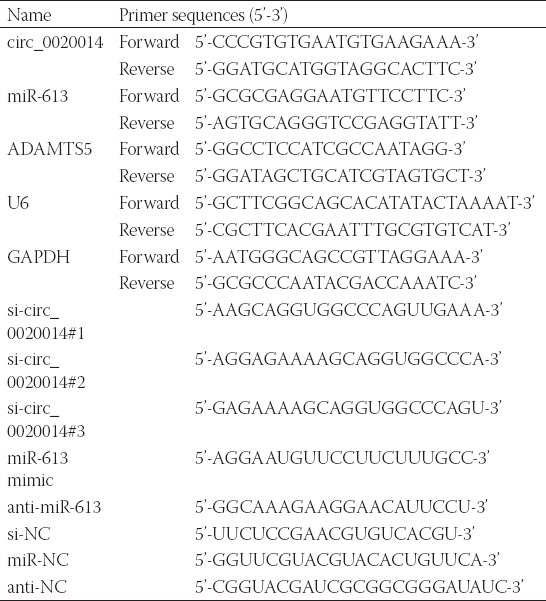
Primers and sequences used in this study.

### Cell transfection

Small interfering RNA against circ_0020014 (si-circ_0020014#1, si-circ_0020014#2, and si-circ_0020014#3), ADAMTS5 overexpressed vector (ADAMTS5), miR-613 mimic (miR-613) or inhibitor (anti-miR-613), and matching controls (si-NC, vector, miR-NC, and anti-NC) were procured from Geneseed Biotech (Guangzhou, China). The oligonucleotides or plasmids were introduced into the cells through Lipofectamine® 2000 reagent (Thermo). After incubation for 24 or 48 hours, the cells were collected for subsequent research. The sequences of these oligonucleotides and siRNA are presented in Table 1.

### 3-(4,5)-dimethylthiahiazo (-z-y1)-2,5-di-phenytetrazoliumromide (MTT) assay

MTT assay was exploited to assess cell viability. 3 × 10^3^ chondrocytes in 200 μL of medium were transplanted into 96-well plates for 48 hours. Then, 20 μL of MTT solution (Life Technologies) was added into each well and incubated with chondrocytes for additional 4 hours. Formazan crystals were dissolved by dimethyl sulfoxide (DMSO; Invitrogen). The absorbance at 570 nm was measured by a microplate reader (BioRad).

### Apoptosis analysis

Transfected chondrocytes were gathered by centrifugation at 1200 × g for 10 minutes and cleaned with phosphate buffer saline 3 times, followed by incubating the cells with Annexin V-FITC and propidium iodide (PI) solution (eBioscience, San Diego, CA, USA) for 10 minutes. The apoptosis rate of chondrocytes was measured with a flow cytometer (FACSort; Becton Dickinson, Franklin Lakes, NJ, USA).

### Western blot assay

Ice-cold RIPA lysis buffer (Beyotime, Shanghai, China) was implemented to isolate total proteins from OA tissues and cell lines. The proteins were then separated by sodium dodecyl sulfate polyacrylamide gel electrophoresis. After that, wet electrophoretic transfer method was performed to transfer protein onto nitrocellulose membranes (BioRad), and then, the membranes were blocked with 5% skimmed milk. After being hatched with specific primary antibody overnight at 4°C, the membranes were then incubated with secondary antibody (#7074S; 1:2000 dilution) for 1.5 hours at room temperature. Finally, the blots were visualized using enhanced chemiluminescence detection kit (Thermo). The specific primary antibodies including anti-cleaved-caspase 3 (#9664S; 1:2000), anti-cleaved-caspase 9 (#20750S; 1:2000), anti-B-cell lymphoma-2 (Bcl-2; #3498S; 1:2000), anti-collagen III (#20750S; 1:2000), anti-matrix metalloprotein13 (MMP13; #69926S; 1:2000), anti-tumor necrosis factor-a (TNF-α; #8184S; 1:2000), anti-IL-6 (IL-6; #12912S; 1:2000), and anti-GAPDH (#2118S; 1:3000) were acquired from Cell Signaling Technology (Danvers, MA, USA), while anti-collagen II (1:5000, ab188570) and anti-ADAMTS5 (1:2000; ab41037) were procured from Abcam (Cambridge, MA, USA).

### Enzyme-linked immunosorbent assay (ELISA)

Chondrocytes were cultured in 24-well plates for 48 hours. Then, liquid supernatant was collected to assess IL-6 and TNF-α levels by commercial ELISA kits (Sigma, San Francisco, CA, USA) following the manufacturer’s protocols. The standard solution and cell supernatant were separately added into the wells of ELISA plates that were pre-coated with IL-6 or TNF-α antibodies and incubated at room temperature for 2 hours. Then, the wells were incubated with secondary antibody conjugated HRP for another 2 hours and then incubated with 100 μL TMB Reagent for 15 minutes in darkness. After the reaction was stopped, the absorbance was measured at 450 nm under the microplate reader (BioRad).

### Dual-luciferase reporter assay

Bioinformatics analysis was accomplished through the online software starBase (http://starbase.sysu.edu.cn/). The circ_0020014 sequence that harbored the wild type (WT) or mutant type (MUT) binding fragments of miR-613 was synthesized and then cloned into the psiCHECK-2 vector (Promega, Madison, WI, USA) to obtain the circ_0020014-WT and circ_0020014-MUT luciferase reporter plasmids. Similarly, luciferase reporter plasmids (ADAMTS5-3’untranslated region (UTR)-WT and ADAMTS5-3’UTR-MUT) were constructed. Approximately 2 × 10^5^ chondrocytes were cointroduced with the luciferase reporter plasmids and miR-613 or miR-NC. Forty-eight hours upon transfection, luciferase activity was tested using Dual-Luciferase Assay Kit (Promega).

### RNA immunoprecipitation (RIP) assay

The RIP assay was conducted using an Imprint^®^ RIP kit (Sigma). The transfected chondrocytes were collected in complete RIP lysis buffer, and cell lysates were interacted with magnetic beads that embraced with anti-Ago2 or anti-IgG at 4°C overnight. Afterward, the samples were incubated with proteinase K to digest protein. Finally, the abundance of circ_0020014 and miR-613 was assessed by RT-qPCR.

### RNA pull-down assay

The 50 nM of 3’end biotinylated miR-613 mimic or control (Ribobio) were transfected into chondrocytes. Forty-eight hours later, cell lysates were hatched with streptavidin C-1 magnetic beads (Invitrogen) for 3 h. The enrichment of circ_0020014 was measured by RT-qPCR.

### Statistical analysis

Three biological replicates were performed for each experiment. Three technical replicates per biological sample were assayed, and mean values were reported. The data were exhibited as mean ± standard deviation by GraphPad Prism 7 (GraphPad, La Jolla, CA, USA). Two-tailed Student’s *t*-test or analysis of variance followed with Bonferroni correction for multiple comparisons. Pearson’s correlation coefficient analysis was used for correlation analyses. *p <* 0.05 was considered statistically significant.

## RESULTS

### Circ_0020014 was upregulated in OA cartilage tissues and IL-1β-treated CHON-001 cells

**FIGURE 1:**
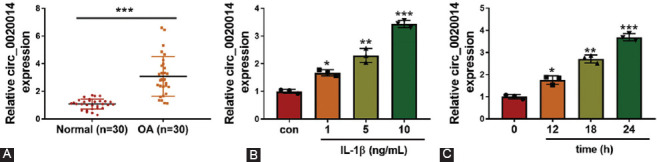
The expression level of circ_0020014 in OA cartilage and IL-1β-treated chondrocytes. (A) The relative expression level of circ_0020014 was determined by RT-qPCR assay in OA cartilage and control. (B-C) After treating with IL-1β, RT-qPCR assay was used to test circ_0020014 expression in chondrocytes. *p < 0.05, **p < 0.01, ***p < 0.001.

First, the expression of circ_0020014 in OA cartilage tissues was measured. The results revealed that circ_0020014 was very clearly upregulated in OA cartilage tissues compared to normal cartilage tissues (Figure 1A). In contrast with the untreated cells, IL-1β treatment could increase the expression of circ_0020014 in CHON-001 cells in a dose- and time-dependent manner (Figure 1B and C). Ten ng/mL IL-1β-treated CHON-001 cells were chosen for subsequent conducted research.

### Knockdown of circ_0020014 promoted cell viability and ECM accumulation but decreased apoptosis in IL-1β-treated CHON-001 cells

**FIGURE 2:**
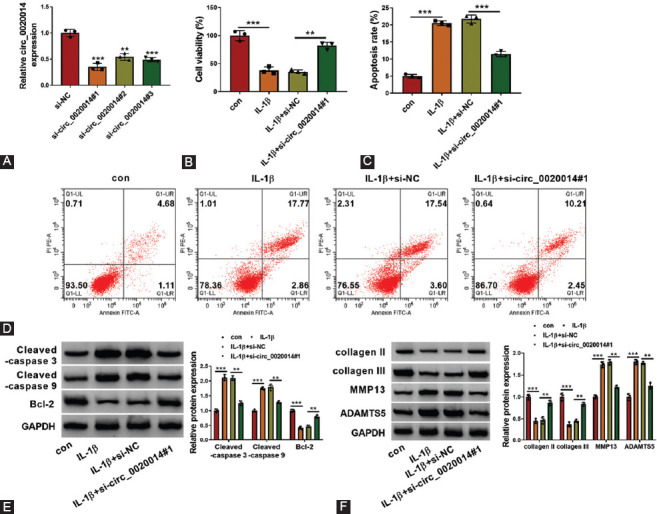
Effects of circ_0020014 silencing on cell viability, apoptosis, and ECM accumulation in IL-1β-treated chondrocytes. (A) The interference efficiency of si-circ_0020014 was assessed by RT-qPCR assay. (B-F) Chondrocytes were divided into four groups: Con, IL-1β, IL-1β+si-NC, and IL-1β+si-circ_0020014. (B) The cell viability was measured by MTT assay. (C-D) The apoptosis rate was measured by flow cytometry assay, and representative pictures were shown. (E-F) The protein expression levels of cleaved-caspase 3, cleaved-caspase 9, Bcl-2, collagen III, MMP13, and ADAMTS5 were quantified by Western blot analysis. **p < 0.01, ***p < 0.001.

To investigate the functional effects of circ_0020014 in OA progression, IL-1β-stimulated CHON-001 cells were transfected with si-circ_0020014#1, si-circ_0020014#2, or si-circ_0020014#3. As shown in Figure 2A, the expression of circ_0020014 was significantly decreased in cells with circ_0020014 knockdown, especially in the si-circ_0020014#1 group. A significant decrease in cell viability was observed in IL-1β-treated CHON-001 cells, which was overturned by knockdown of circ_0020014 (Figure 2B). Furthermore, the inhibition of circ_0020014 protected CHON-001 cells from IL-1β-induced apoptosis (Figure 2C and D). In addition, the protein levels of apoptosis markers cleaved-caspase 3 and cleaved-caspase 9 were upregulated, while the level of pro-apoptotic protein Bcl-2 was downregulated in IL-1β-treated CHON-001 cells, while silencing of circ_0020014 counteracted these effects (Figure 2E). Degradation of ECM proteins has been considered as an important pathological characteristic of OA [18]. MMP13 and ADAMTS5 are critical regulators involved in the regulation of OA development by cleaving type II collagen or degrading aggrecan in articular cartilage [19]. As shown in Figure 2F, treatment with IL-1β elevated the protein levels of ECM degradation-associated proteases (MMP13 and ADAMTS5), but suppressed the levels of ECM proteins (collagen II and collagen III) in CHON-001 cells, which were overturned by suppression of circ_0020014. Hence, circ_0020014 knockdown impeded the development of OA by regulating cell viability, apoptosis, and ECM accumulation in IL-1β-treated CHON-001 cells.

### Circ_0020014 silencing inhibited inflammation in IL-1β-treated CHON-001 cells

**FIGURE 3:**
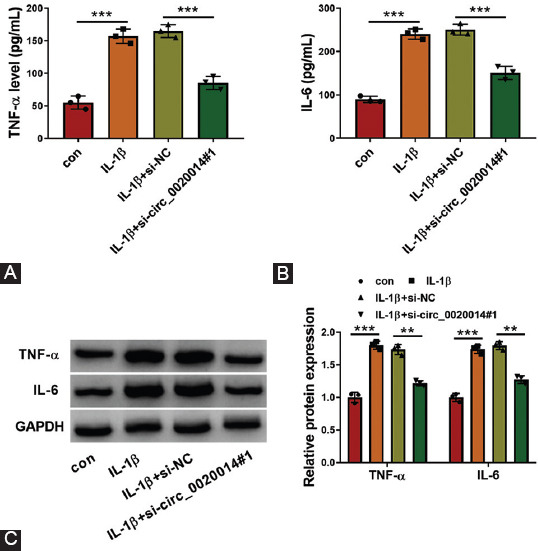
Effects of circ_0020014 inhibition on inflammation in IL-1β-treated chondrocytes. (A-B) The supernatant abundance of TNF-α and IL-6 was analyzed by matched kits in IL-1β-treated chondrocytes transfected with si-NC or si-circ_0020014#1, with untreated cells as con group. (C) The protein expression levels of TNF-α and IL-6 were assessed by Western blot analysis in IL-1β-treated chondrocytes transfected with si-NC or si-circ_0020014#1, with untreated cells as con group. **p < 0.01, ***p < 0.001.

As presented in Figure 3A and B, the secretion of TNF-α and IL-6 in CHON-001 cells was promoted by IL-1β treatment, whereas this effect was abolished by silencing of circ_0020014 in IL-1β-treated CHON-001 cells. Consistently, Western blot analysis also suggested that transfection of si-circ_0020014#1 partly overturned the promotion effects of IL-1β treatment on the protein levels of TNF-α and IL-6 in CHON-001 cells (Figure 3C). Therefore, circ_0020014 downregulation inhibited inflammation in IL-1β-treated CHON-001 cells.

### Circ_0020014 acted as a sponge for miR-613 in IL-1β-treated CHON-001 cells

**FIGURE 4:**
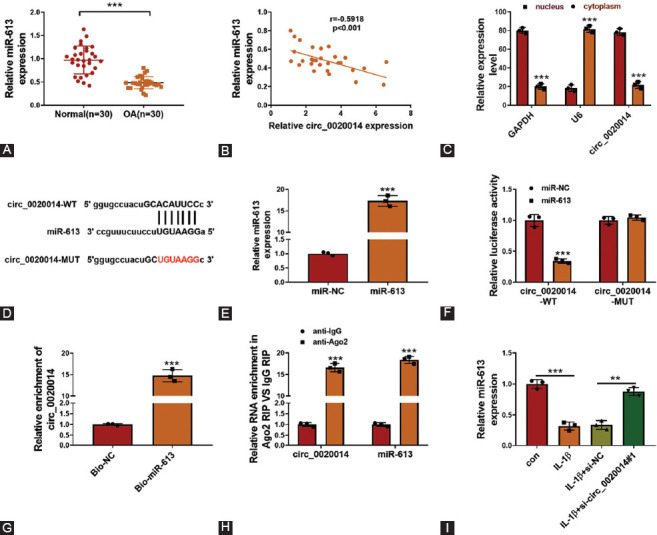
MiR-613 was a direct target of circ_0020014. (A) The expression level of miR-613 was examined by RT-qPCR assay in OA cartilage and control. (B) The correlation relationship between the levels of miR-613 and circ_0020014 was analyzed by Pearson’s correlation analysis. (C) The subcellular localization of circ_0020014 in chondrocytes was analyzed by subcellular fractionation experiment. (D) The complementary sequences between miR-613 and circ_0020014 were shown. (E) The expression level of miR-613 was assessed by RT-qPCR assay in chondrocytes transfected with miR-613 or miR-NC. (F-H) The association between miR-613 and circ_0020014 was analyzed by dual-luciferase reporter, RNA pull-down, and RIP assays. (I) RT-qPCR assay was used to test miR-613 expression in IL-1β-treated chondrocytes transfected with si-NC or si-circ_0020014#1. **p < 0.01, ***p < 0.001.

**SUPPLEMENTARY FIGURE 1:**
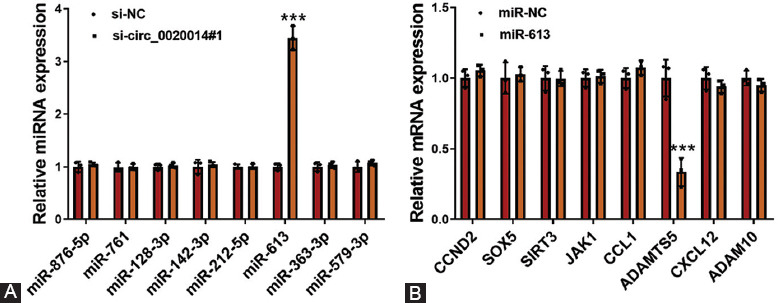
The potential targets of circ_0020014 and miR-613 in chondrocytes. (A) The expression levels of miR-876-5p, miR-761, miR-128-3p, miR-142-3p, miR-212-5p, miR-613, miR-363-3p, and miR-579-3p in chondrocytes with si-NC or si-circ_0020014#1 transfection were detected by RT-qPCR assay. (B) The expression levels of CCND2, SOX5, SIRT3, JAK1, CCL1, ADAMTS5, CXCL12, and ADAM10 in chondrocytes with miR-NC or miR-613 mimic transfection were detected by RT-qPCR assay. ***p < 0.001.

Using starBase software, several miRNAs were predicted to be the target of circ_0020014. Among these miRNAs, miR-613 was the most upregulated miRNA in IL-1β-treated CHON-001 cells with circ_0020014 knockdown (Supplementary Figure 1A), which was chosen for subsequent research. MiR-613 was downregulated in OA cartilage tissues compared to in normal control (Figure 4A) and was negatively correlated with circ_0020014 expression in OA cartilage tissues (Figure 4B). Subcellular fractionation experiment disclosed that circ_0020014 is mainly located in the cytoplasm of IL-1β-treated CHON-001 cells (Figure 4C), indicating that circ_0020014 may act as miRNA sponges. The complementary binding sequences between miR-613 and circ_0020014 are presented in Figure 4D. RT-qPCR analysis revealed that miR-613 was significantly upregulated by miR-613 transfection in IL-1β-treated CHON-001 cells (Figure 4E). Dual-luciferase reporter assay demonstrated that miR-613 overexpression decreased the luciferase activity of circ_0020014-WT group, but not circ_0020014-MUT group (Figure 4F). RNA pull-down assay revealed that circ_0020014 was enriched by Bio-miR-613 in CHON-001 cells compared with control group (Figure 4G). Moreover, circ_0020014 and miR-613 were significantly enriched in anti-Ago2 group, rather than anti-IgG group (Figure 4H), which confirmed the correlation between circ_0020014 and miR-613. In addition, downregulation of miR-613 in IL-1β-treated CHON-001 cells was reversed by inhibition of circ_0020014 (Figure 4I). Collectively, miR-613 was a direct target of circ_0020014.

### Circ_0020014 regulated cell viability, apoptosis, ECM accumulation, and inflammation in IL-1β-treated CHON-001 cells by sponging miR-613

**FIGURE 5:**
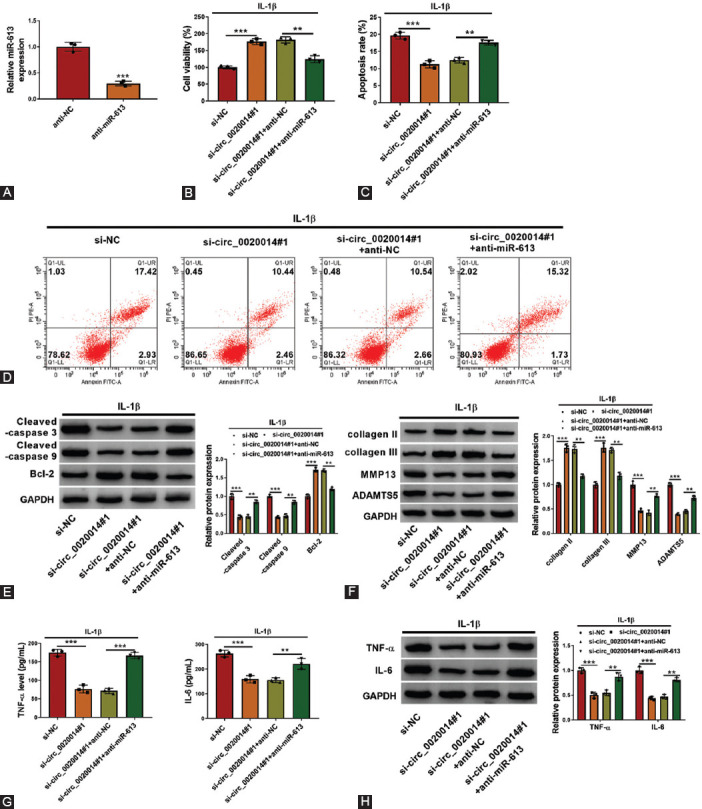
Knockdown of circ_0020014-mediated effects on chondrocytes was abolished by silencing miR-613. (A) RT-qPCR was used to confirm the knockdown efficiency of anti-miR-613 in chondrocytes. (B-H) IL-1β-treated chondrocytes were transfected with si-NC, si-circ_0020014#1, si-circ_0020014#1+anti-NC, or si-circ_0020014#1+anti-miR-613. (B) MTT assay was performed for examining the cell viability. (C-D) Flow cytometry assay was performed to test apoptosis rate. (E-F) The protein expression levels were quantified by Western blot analysis in chondrocytes. (G) The inflammation in IL-1β-treated chondrocytes was assessed by checking supernatant abundance of TNF-α and IL-6 by ELISA. (H) Western blot analysis was conducted to quantify the expression of TNF-α and IL-6 in chondrocytes. **p < 0.01, ***p < 0.001.

The association between circ_0020014 and miR-613 was investigated in IL-1β-treated CHON-001 cells. We found that miR-613 was downregulated in anti-miR-613-transfected CHON-001 cells compared with control (Figure 5A). MTT assay revealed that cell viability was enhanced by circ_0020014 knockdown in IL-1β-treated CHON-001 cells, which was rescued by cotransfection of anti-miR-613 (Figure 5B). Inhibition of circ_0020014 repressed cell apoptosis in IL-1β-treated CHON-001 cells, whereas suppression of miR-613 blocked this effect (Figure 5C and D). In addition, circ_0020014 silencing significantly enhanced Bcl-2 protein level but decreased cleaved-caspase 3 and cleaved-caspase 9 protein levels, while cotransfection of miR-613 inhibitor partly overturned theses effects in IL-1β-treated CHON-001 cells (Figure 5E). Promotion effects on collagen II and collagen III protein levels, as well as the inhibitory effects on the protein levels of MMP13 and ADAMTS5 in IL-1β-treated CHON-001 cells, which were mediated by silencing circ_0020014, were abolished by downregulation of miR-613 (Figure 5). In addition, we observed that circ_0020014 silencing inhibited inflammatory reaction by decreasing TNF-α and IL-6 levels, while cotransfection of miR-613 inhibitor prominently reversed these changes (Figure 5G and H). Hence, circ_0020014 mediated IL-1β-treated CHON-001 cell injury by sponging miR-613.

### MiR-613 targeted ADAMTS5 in IL-1β-treated CHON-001 cells

**FIGURE 6:**
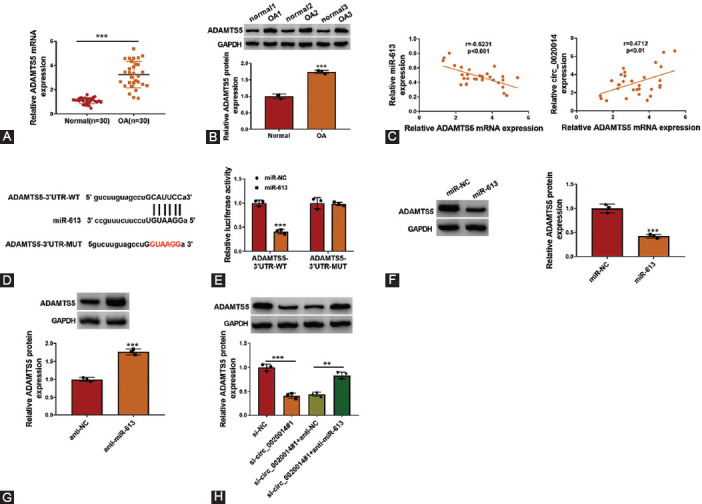
ADAMTS5 was a functional gene of miR-613. (A-B) RT-qPCR and Western blot assay were used to examine ADAMTS5 levels in OA cartilage and control. (C) The correlation relationships between the levels of ADAMTS5 and miR-613 or circ_0020014 were analyzed by Pearson’s correlation analysis. (D) The binding regions between ADAMTS5 and miR-613 were shown. (E) Dual-luciferase reporter assay was performed in transfected chondrocytes. (F-H) The protein expression of ADAMTS5 was assessed by Western blot assay in chondrocytes transfected with miR-613 mimic or inhibitor, along with in IL-1β-treated chondrocytes transfected with si-NC, si-circ_0020014#1, si-circ_0020014#1+anti-NC, or si-circ_0020014#1+anti-miR-613. **p < 0.01, ***p < 0.001.

Bioinformatics analysis revealed several potential mRNAs that might be targeted by miR-613. We found that miR-613 exhibited the strongest inhibitory effect on ADAMTS5 since it was the most downregulated mRNA in IL-1β-treated CHON-001 cells with miR-613 overexpression (Supplementary Figure 1B). As shown in Figure 6A and B, the mRNA and protein levels of ADAMTS5 were upregulated in OA cartilage tissues. Furthermore, correlation analysis confirmed that the expression of ADAMTS5 was negatively correlated with miR-613 expression, but was positively correlated with circ_0020014 expression in OA cartilage tissues (Figure 6C). The binding regions between ADAMTS5 and miR-613 are presented in Figure 6D. Dual-luciferase reporter assay indicated that miR-613 mimic transfection reduced the luciferase activity of ADAMTS5-3’UTR-WT group, while luciferase activity of ADAMTS5-3’UTR-MUT group was not affected by miR-613 overexpression (Figure 6E). The protein level of ADAMTS5 was downregulated by overexpression of miR-613 in IL-1β-treated CHON-001 cells, but was upregulated by transfection of miR-613 inhibitor (Figure 6F and G). Circ_0020014 knockdown inhibited the protein level of ADAMTS5 in IL-1β-treated CHON-001 cells, whereas this effect was abolished by co-transfection of miR-613 inhibitor (Figure 6H). In summary, ADAMTS5 was a direct target of miR-613.

### Overexpression of ADAMTS5 reversed circ_0020014 knockdown induced the effect in IL-1β-treated CHON-001 cells

**FIGURE 7:**
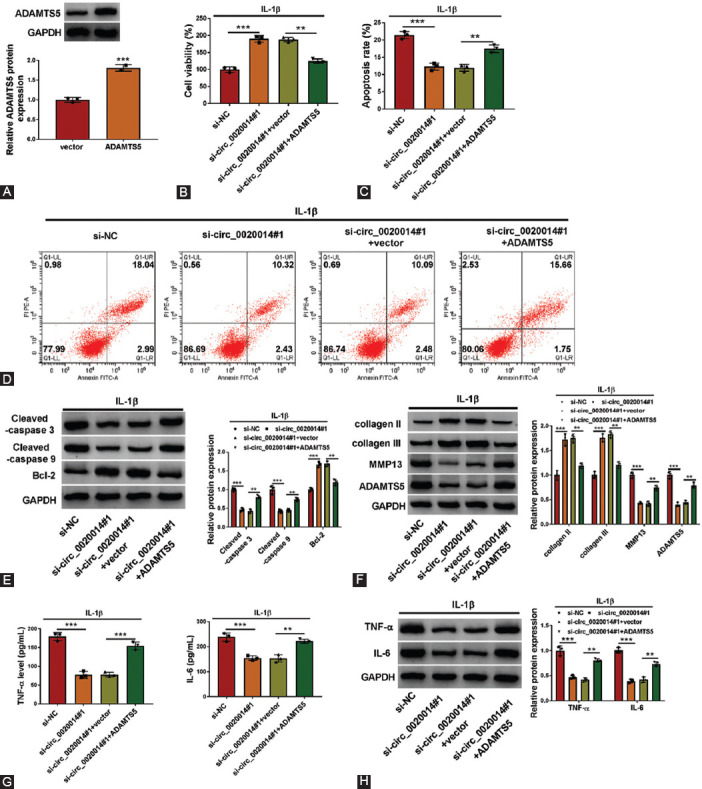
Circ_0020014 regulated cell viability, apoptosis, ECM accumulation, and inflammation in IL-1β-treated chondrocytes by regulating ADAMTS5. (A) Western blot analysis was used to determine the expression of ADAMTS5 in chondrocytes transfected with ADAMTS5 or vector. (B-H) IL-1β-treated chondrocytes were transfected with si-NC, si-circ_0020014#1, si-circ_0020014#1+vector, or si-circ_0020014#1+ADAMTS5. (B) The cell viability was calculated by MTT assay. (C-D) The apoptosis rate was estimated by flow cytometry assay. (E-F) The protein expression levels of cleaved-caspase 3, cleaved-caspase 9, Bcl-2, collagen III, MMP13, and ADAMTS5 were evaluated by Western blot analysis. (G) The supernatant abundance of TNF-α and IL-6 was detected by kits. (H) The expression of TNF-α and IL-6 was measured by Western blot analysis in chondrocytes. **p < 0.01, ***p < 0.001.

To analyze the roles of ADAMTS5 and its correlation with circ_0020014 in IL-1β-treated CHON-001 cells, a set of rescue experiments were conducted in IL-1β-treated CHON-001 cells. As shown in Figure 7A, transfection of ADAMTS5 overexpression vector enhanced the expression of ADAMTS5 in IL-1β-treated CHON-001 cells. ADAMTS5 overexpression reversed the proliferative effect of circ_0020014 inhibition in IL-1β-treated CHON-001 cells (Figure 7B). Upregulation of ADAMTS5 overturned the inhibitory effect of circ_0020014 knockdown on cell apoptosis in IL-1β-treated CHON-001 cells (Figure 7C and D). Furthermore, silencing of circ_0020014 increased the expression of Bcl-2, collagen II, and collagen III, but decreased cleaved-caspase 3, cleaved-caspase 9, MMP13, and ADAMTS5 protein levels in IL-1β-treated CHON-001 cells, whereas these effects were overturned by overexpression of ADAMTS5 (Figure 7E and F). In addition, ADAMTS5 overexpression rescued the suppressive effects of circ_0020014 knockdown on inflammation in IL-1β-treated CHON-001 cells by increasing the secretion of TNF-α and IL-6 (Figure 7G and H). In conclusion, circ_0020014 could regulate cell viability, apoptosis, ECM accumulation, and inflammation in CHON-001 cells by regulating ADAMTS5.

## DISCUSSION

In the progression of OA, disequilibrium of ECM accumulation was deemed as the dominant phenotype in dysfunctional chondrocytes, which could be triggered by pro-inflammatory factors [20,21]. IL-1β, a type of pro-inflammatory cytokine, was widely used to induce cartilage matrix degradation in OA [22,23], and thus chondrocytes treated with IL-1β could be used to mimic OA chondrocytes *in vitro* [24,25]. Previous reports had revealed that pro-inflammatory cytokines could inhibit proliferation and induce apoptosis in chondrocytes [26]. The enhancement of ECM degradation could accelerate the progression of OA [27]. In our research, IL-1β treatment inhibited cell viability and ECM accumulation, but promoted apoptosis and inflammation in chondrocytes. Therefore, IL-1β-treated human CHON-001 cells could be used as the *in vitro* cellular model for OA.

CircRNAs play a crucial function in the development of OA by regulating ECM degradation, inflammation, and apoptosis [28]. A recent study has shown that circ_0020014 is differentially expressed in OA and Kashin-Beck disease patients, which may be a potential target for the diagnosis of the two diseases [6]. In our research, an obvious increase in circ_0020014 expression was found in OA cartilage specimens and IL-1β-induced CHON-001 cells. Circ_0020014 knockdown significantly increased cell viability and ECM accumulation but inhibited apoptosis and inflammation in IL-1β-treated CHON-001 cells, indicating that circ_0020014 might be a potential target for OA therapy.

Numerous studies have disclosed that miRNAs are closely associated with the development of OA [29]. Several miRNAs, including miR-93 [30], miR-181a-5p [31], and miR-140-5p [32], were suggested to participate in OA pathogenesis and could be used as a promising diagnostic and therapeutic target for OA. In the present research, miR-613 was predicted and confirmed to be the target of circ_0020014 in IL-1β-treated CHON-001 cells. MiR-613 is a well-explored miRNA in cancers and diseases [33-35]. However, few studies have examined the function of miR-613 in OA. A recent research showed that miR-613 relieved IL-1β-induced cell injury and inflammatory response in chondrocytes by targeting fibronectin 1 [36]. In the present research, miR-613 was downregulated in OA, and its level was adversely associated with circ_0020014 level in OA cartilage tissues. MiR-613 downregulation could overturn the effect of circ_0020014 silence on IL-1β-treated CHON-001 cell injury. Circ_0020014 exerted its function in IL-1β-treated CHON-001 cells by sponging miR-613, and miR-613 functioned as a protective effect in OA.

**FIGURE 8:**
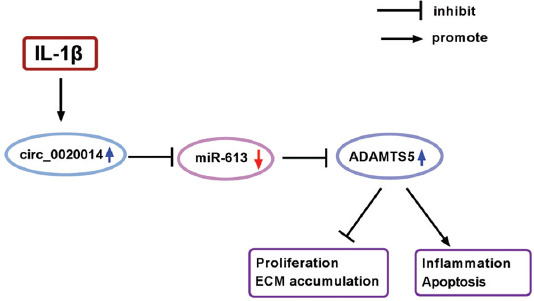
Mechanism diagram for our research. Circ_0020014 regulated cell viability, apoptosis, ECM accumulation, and inflammation in IL-1β-treated chondrocytes by the miR-613/ADAMTS5 axis.

Accumulating evidence provided that miRNAs regulate gene expression by targeting 3’UTR of target mRNAs to regulate mRNA degradation or translational inhibition [37]. Our results supported that ADAMTS5 was targeted by miR-613 in chondrocytes. Several miRNAs, such as miR-140 [38], miR-21-5p [39], and miR-105 [40], have been reported to be implicated to target ADAMTS5 and then regulate ECM homeostasis in OA progression. MiR-145 was suggested to increase chondrocytes viability and inhibit cartilage ECM degradation in IL-1β-incubated chondrocytes by targeting ADAMTS5 [41]. Upregulation of miR-27a-3p decreased ADAMTS5 expression in chondrocytes caused by IL-1β [42]. These results showed that ADAMTS5 could be a therapeutic biomarker for OA. Herein, ADAMTS5 was overexpressed in OA cartilage specimens and cells. We confirmed that circ_0020014 could regulate the expression of ADAMTS5 by competitively binding to miR-613. In addition, overexpressed ADAMTS5 partly overturned the functions of circ_0020014 knockdown in OA development. Altogether, circ_0020014 could regulate IL-1β-treated CHON-001 cell damage by mediating miR-613/ADAMTS5 axis (Figure 8).

However, the possible function and regulatory mechanism of circ_0020014/miR-613/ADAMTS5 axis were confirmed only at the cellular level. Although the *in vitro* cellular model is suitable for mechanistic studies, the *in vivo* experiments are closer to physiological conditions and are crucial to determine the reliability of our results. To further confirm our findings, *in vivo* animal and clinical research are needed in future research.

## CONCLUSION

In summary, circ_0020014 level was elevated in OA cartilage specimens and IL-1β-treated CHON-001 cells. Circ_0020014 could inhibit cell viability and ECM accumulation but promote apoptosis and inflammation in IL-1β-treated CHON-001 cells by regulating miR-613/ADAMTS5 axis. Altogether, our research identified the critical role of circ_0020014/miR-613/ADAMTS5 axis in OA development, which might provide novel therapeutic target for OA.
